# The sperm of aging male bustards retards their offspring's development

**DOI:** 10.1038/ncomms7146

**Published:** 2015-02-03

**Authors:** Brian T. Preston, Michel Saint Jalme, Yves Hingrat, Frederic Lacroix, Gabriele Sorci

**Affiliations:** 1 UMR CNRS/uB 6282 Biogeosciences, Universite de Bourgogne, 6 bd Gabriel, 21000 Dijon, France; 2 UMR 7204 CERSP, Museum National d'Histoire Naturelle, 57 rue Cuvier, 75005 Paris, France; 3 RENECO Wildlife Preservation, PO Box 61741, Abu Dhabi, United Arab Emirates

## Abstract

Understanding whether the sperm of older males has a diminished capacity to produce successful offspring is a key challenge in evolutionary biology. We investigate this issue using 10 years of reproductive data on captive long-lived houbara bustards (*Chlamydotis undulata*), where the use of artificial insemination techniques means parents can only influence offspring quality via their gametes. Here we show that paternal aging reduces both the likelihood that eggs hatch and the rate at which chicks grow, with older males producing the lightest offspring after the first month. Surprisingly, this cost of paternal aging on offspring development is of a similar scale to that associated with maternal aging. Fitting with predictions on germline aging, the sperm of immature males produce the fastest growing offspring. Our findings thus indicate that any good genes benefit that might be offered by older ‘proven' males will be eroded by aging of their germline DNA.

## Abstract (web summary)

Sperm from aging males may decline in quality, but it is unclear how aging affects the ability of males to produce successful offspring. Here, the authors show that paternal aging of captive long-lived houbara bustards reduces both the likelihood that eggs hatch and the rate at which chicks grow.

## Graphical abstract

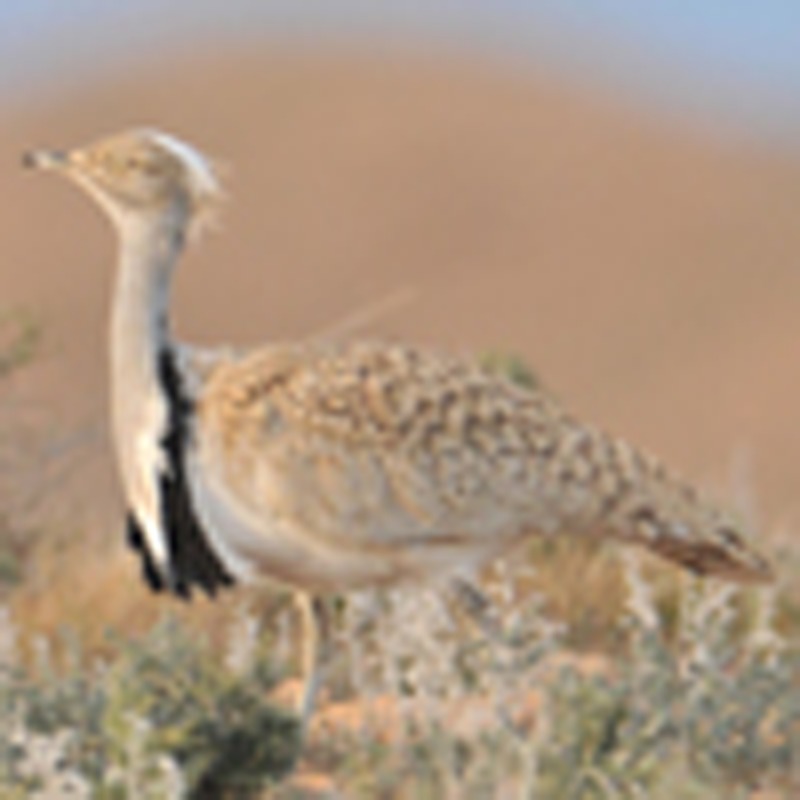

Senescence is the deterioration in physical condition that occurs in aging animals^1^. In nature, senescence is increasingly being recognized as a widespread constraint on lifetime reproductive output^2^, degrading multiple fitness traits and potentially generating complex patterns of evolutionary conflict between the sexes^3^,^4^,^5^. In male vertebrates, traits associated with reproductive competitiveness have been found to decline in aged males (for example, rutting activity^6^, social dominance^7^, sexual signalling^8^), with the expectation that this will lead to lower reproductive output via competitive exclusion or female mating biases. Far less considered is the possible influence that male aging may have on their post insemination success through declines in the viability of their gametes, and most notably, the intrinsic quality of offspring their gametes can produce.

Gametes from aging males may undergo senescent declines in quality via two principal mechanisms. First, there can be a progressive deterioration in the performance of males' spermatogenic machinery as they age, associated with declines in the levels of reproductive hormones, and resulting in sperm that appear less able to deliver their genetic load to female ova^9^. Perhaps more importantly, however, it is also expected that there is an increasing probability of mutation within a male's germline over time, associated with the number of stem cell divisions during spermatogenesis, and resulting in a degradation of the DNA that is carried within their gametes^10^,^11^. Evidence is beginning to emerge from longitudinal studies that sperm function can decline in aging vertebrate males^12^, though the degree to which senescent declines in the functional performance or genetic integrity of gametes might impinge on the overall viability and quality of progeny is not known.

Here we examine the post insemination success of long-lived male houbara bustards (*Chlamydotis undulata*) that have been part of a large-scale conservation programme in Eastern Morocco. Importantly, the aim of this study is that the conservation programme utilizes artificial insemination and incubation followed by hand-rearing of hatched chicks. Thus, critically, parental influence is achieved only via their gametes, with females neither being able to choose prospective sires nor alter their allocation of care based on differing assessments of inseminating males or the quality of offspring produced^5^,^13^. Ten years of longitudinal records have been collected on reproductive parameters of >1,000 different males and females ranging from 1 to 23 years of age. This conservation programme thus provides a unique opportunity for large-scale longitudinal analyses on the reproductive consequences of gametic senesence in a wild long-lived vertebrate. In total, the viability of 58,977 eggs and developmental data on 31,404 chicks have been used in mixed model analyses that were aimed at testing the influence of male gametic aging on both the viability and quality of their offspring. Any evidence of reduced offspring viability and/or quality produced by older males would be indicative of senescent declines in the reproductive performance of their gametes, but the predictions for the nature of the relationship during adolescence would differ depending upon the dominant mechanism of sperm aging, that is, whether it is due to senescence of the spermatogenic machinery or of germline integrity. A previous study on this species found that measures of ejaculate quality (the number, motility and % aberrant morphology of sperm) rise through maturation up to 4 years of age before undergoing senescent declines^12^, thus we expect that patterns of egg viability (as determined by hatching rate) will mirror this relationship if aging of male spermatogenic machinery—and hence the functional performance of their sperm—hinders their ability to produce viable offspring. However, if mutation-based aging of gametic DNA is the dominant mechanism of senescent decline, we would instead predict reductions in both offspring viability and subsequent development from the onset of spermatogenesis, as this is the point at which harmful mutations may begin to accumulate in the germline. Thus, the sperm of young males would be expected to produce more viable zygotes and the highest quality offspring. In addition to these predicted relationships, we test and statistically control for an expected and possibly confounding influence of female aging on the quality of eggs and offspring^14^, while also controlling for changes that arise from yearly, seasonal, laying order effects and individual viability^15^. Our results show that paternal aging affects both the viability of eggs and the growth rate of offspring, ultimately resulting in lighter chicks at 30 days of age. These findings are consistent with the mutation-based aging of male germ line DNA, and suggest that any benefit females may gain through mating with older ‘proven' males must be offset against the cost of utilizing the aging DNA that their sperm carry.

## Results

### Hatching success

**Figure 1:**
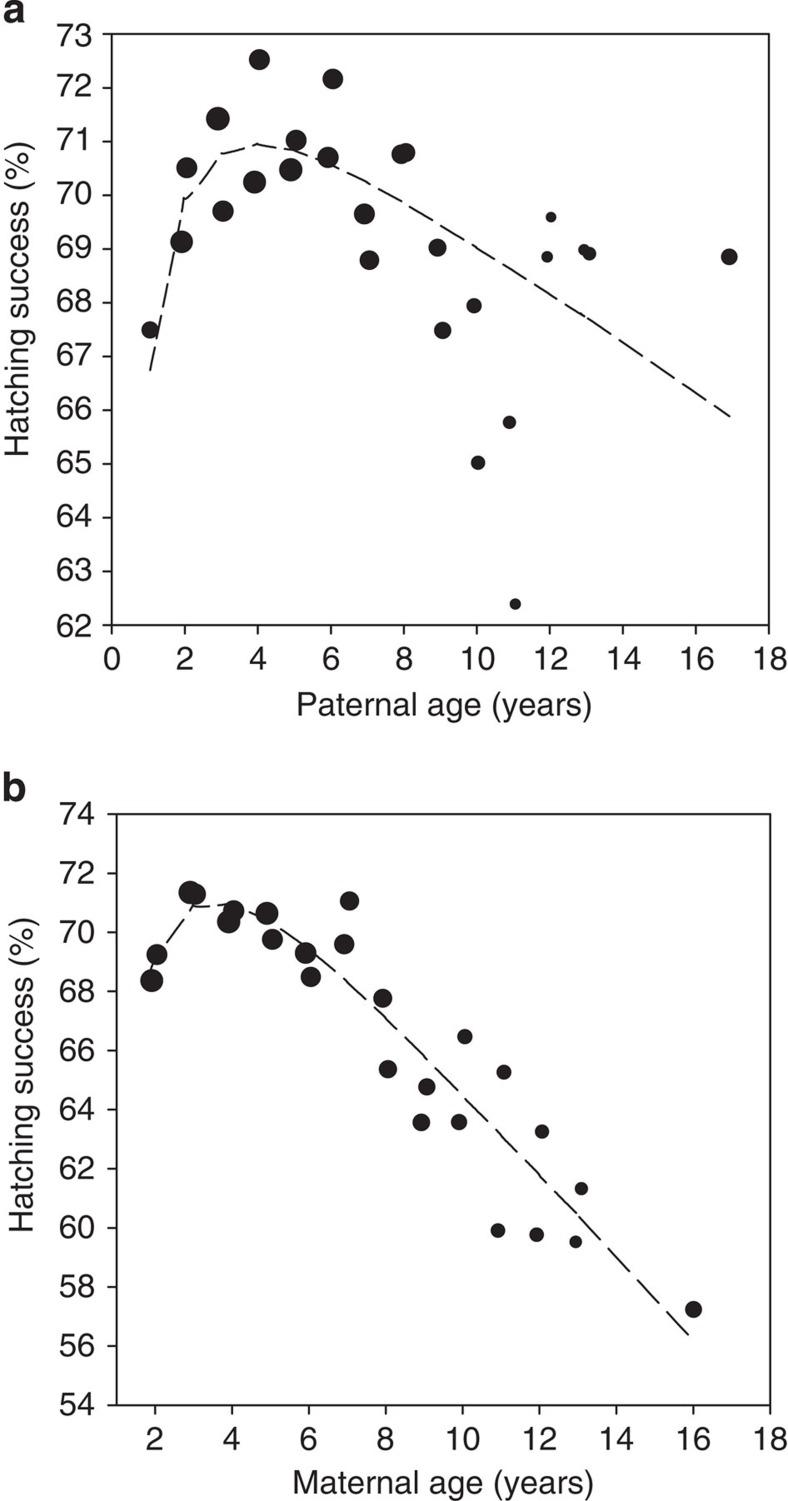
The role of parental aging in the hatching success of eggs. The plots illustrate how hatching success varies according to (**a**) the age of the male whose sperm was used to inseminate the female and (**b**) the age of the female that laid the egg. For both parents, hatching success increases to peak levels at ∼4 years of age before undergoing a senescent decline. Note the differences in scale on the *y*-axis for the different sexes, indicating a greater influence of female age. For illustrative purposes, the data are grouped at 6-month intervals and from 13.5 years of age after which sample sizes are smaller. Data point size reflects the (logged) sample size in each group.

We firstly implemented a Generalized Linear Mixed Model (GLMM) analysis to examine how paternal aging influenced the hatching success of eggs that were produced after their sperm had been used to artificially inseminate females. While statistically controlling for seasonal and laying order effects on eggs, hatching success increased up to a peak paternal age of ∼3 to 4 years, before showing clear evidence of decline beyond 6 years of age (Table 1; Fig. 1a). This pattern mirrors the changes in male ejaculate quality with age that have already been observed in this species^12^, suggesting that they are due to the maturation and then the senescence of males' spermatogenic machinery. Similar patterns of senescence were also present for mothers (Table 1; Fig. 1b), though the rate of decline in hatching success appeared more rapid for females, declining from a model estimated peak of around 71% at 4 years of age for both parents (calculated when matched with peak age partners), to around 66% and 56% for eggs laid 10 years later in the lives of males and females, respectively. Differences in the rate at which male ejaculates collected for the programme has the potential to confound our analyses since it may lead to either the accumulation of ‘old' subfertile spermatids in males whose ejaculates are collected infrequently^16^, or may lead to a depletion in the number of sperm available for insemination from males whose ejaculates are collected frequently^17^. However, changes in hatching success were unrelated to the number of sperm artificially inseminated into females (*P*>0.3 when added to the GLMM model in Table 1, *n*_eggs_=18,127), and were not influenced by the collection frequency of donor males (the number of days since the donor male's last ejaculate and the cumulative number of ejaculates produced by the donor male that season had *P*>0.1 and 0.4 respectively when tested in the GLMM model in Table 1, *n*_eggs_=56,983). Thus, the observed age-related declines in hatching success were not due to a transient depletion or aging of male ejaculatory components that might be caused by differences in their recent copulatory history^17^.

### Offspring mass at hatching

**Figure 2:**
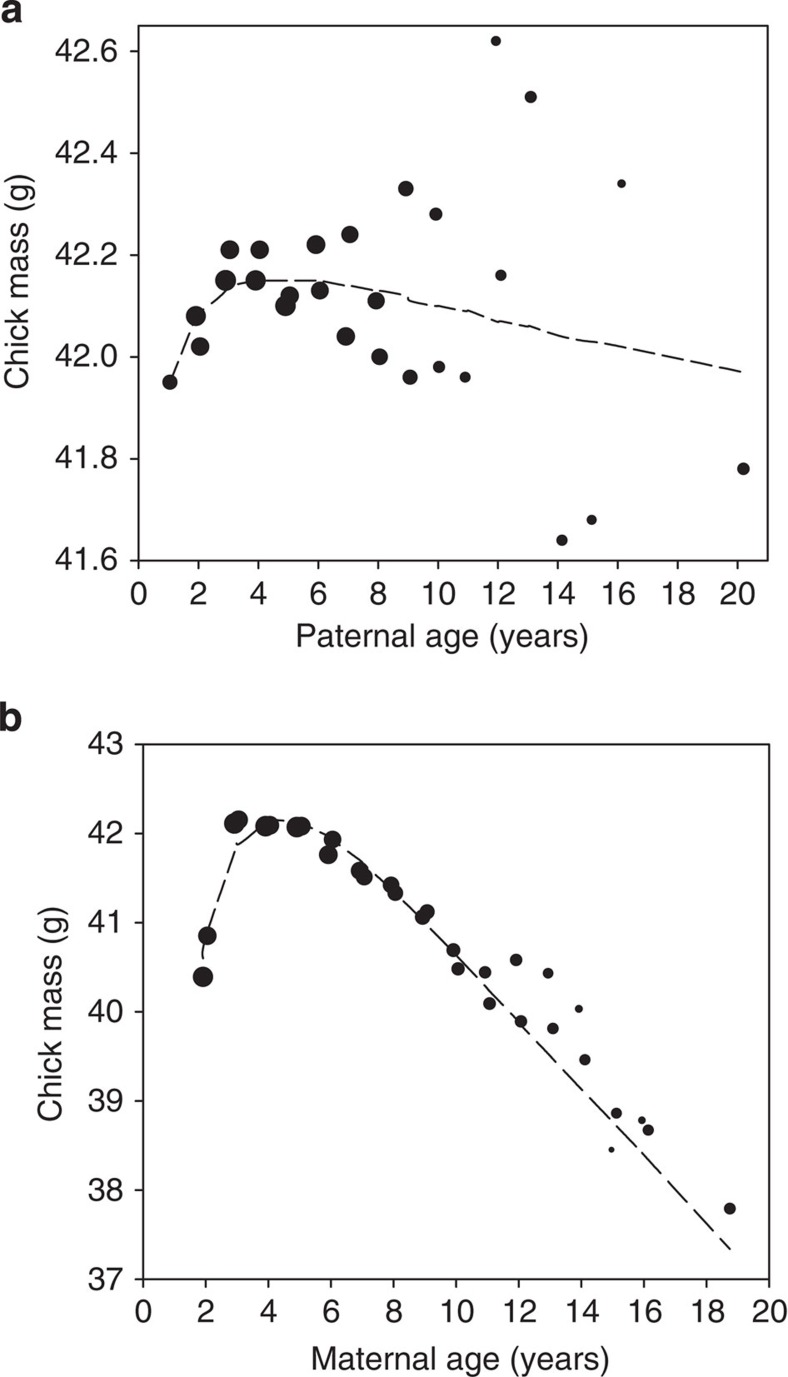
Parental aging and the mass of their offspring at hatching. The plots show how hatchling mass varies according to (**a**) paternal age and (**b**) maternal age. The mass of hatchlings peaks with parents of ∼4 years of age, before declining in older parents. Note that there are large differences in scale between the plots, indicating that maternal age has much greater influence on the hatching mass of offspring than the age of the siring male. For illustrative purposes, the data are grouped at 6-month intervals up to 16 years of age, after which remaining ages are grouped owing to reduced sample size. The size of data points reflects the (logged) sample size in each group.

We next used a linear mixed model (LMM) analysis to test for an influence of paternal age on the hatching mass of their chicks. The results indicated that there was no significant influence of paternal aging, with estimates from the model suggesting that hatching mass of offspring may change by <0.5% across the lifetime of males considered here (Table 2; Fig. 2a). In marked contrast to paternal aging, our analysis further emphasized the importance of maternal senescence on reproductive performance (see Table 2). As was found with the hatching success of eggs, the mass of newly-hatched chicks rose to a peak when mothers were ∼3 to 6 years of age, before undergoing a marked decline (Fig. 2b). The magnitude of this decline was such that when mothers had reached 16 years of age, there was a corresponding 8.3% reduction in the hatching mass of their chicks compared with when they were at their peak ages. This analysis controlled for apparent differences in egg quality that arose from both seasonal effects and the order in which they were laid within and between clutches, precluding these factors as possible explanations for our findings (see Table 2). Thus, maternal senescence appears to have a pronounced effect on the hatching mass of their offspring, while paternal age appears to have little or no influence in this species.

### Offspring growth

**Figure 3:**
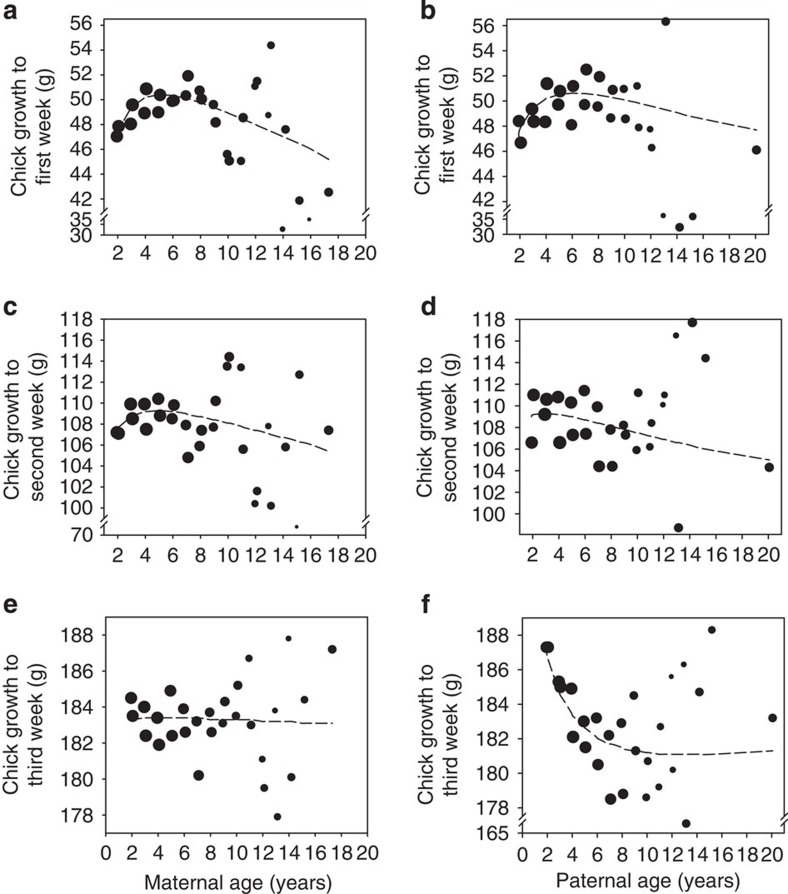
A changing influence of parental age on early offspring growth. The plots show the growth of chicks within the first month of life with respect to the age of their parents, providing a snapshot of growth in the (**a**,**b**) first, (**c**,**d**) second and (**e**,**f**) third week after hatching. Note that hatching masses are equalized (to a mean value) and the plots illustrate only differences that emerge between chicks post hatching. For illustrative purposes, plots contain data only for the week they represent, and the data are grouped at 6-month intervals up to 16 years of age, after which all remaining ages are grouped due to lower sample size. Note that the size of data points reflects the logged sample size in each group and thus sample sizes are considerably larger for younger age groups.

Finally, we used an LMM to analyze the influence that paternal aging might have on the ontogeny of hatched chicks by following growth across their first month of life (that is post hatching), utilizing the repeated measurements of each individual's mass that were collected during this period (∼13 measurements per chick, see the methods). We controlled for the effects of differing seasons on early growth in our model, and to prevent this analysis reflecting the factors influencing the pre-hatching differences in chick development that have already been identified (see above) we also controlled for ‘chick mass at hatching' within our model (see Table 3). Thus, importantly, the analysis focuses only on post-hatching growth. Our model revealed highly significant interactions between stage of development and the age of each parent in determining offspring mass, which indicates that chick growth trajectories differed depending on both paternal and maternal age (chick age × parent age interactions; see Table 3). On closer examination, the relationships predicted by the model are such that early growth (in the first week) appears to be broadly consistent with the pre-hatching developmental benefits of ‘peak age' parents of both sexes (Fig. 3a,b). However, this advantage rapidly disappears as the chicks grow, resulting in no apparent advantage associated with maternal age by week 3 of development, and a new pattern emerging for chicks sired by fathers of different ages, with those born to the youngest fathers experiencing the greatest overall growth up to this period (see Fig. 3c–f). Thus, increasing paternal age appeared to inhibit the growth of offspring during their first month of life, whereas chick development appeared to be unaffected overall by the age of their mothers.

**Figure 4:**
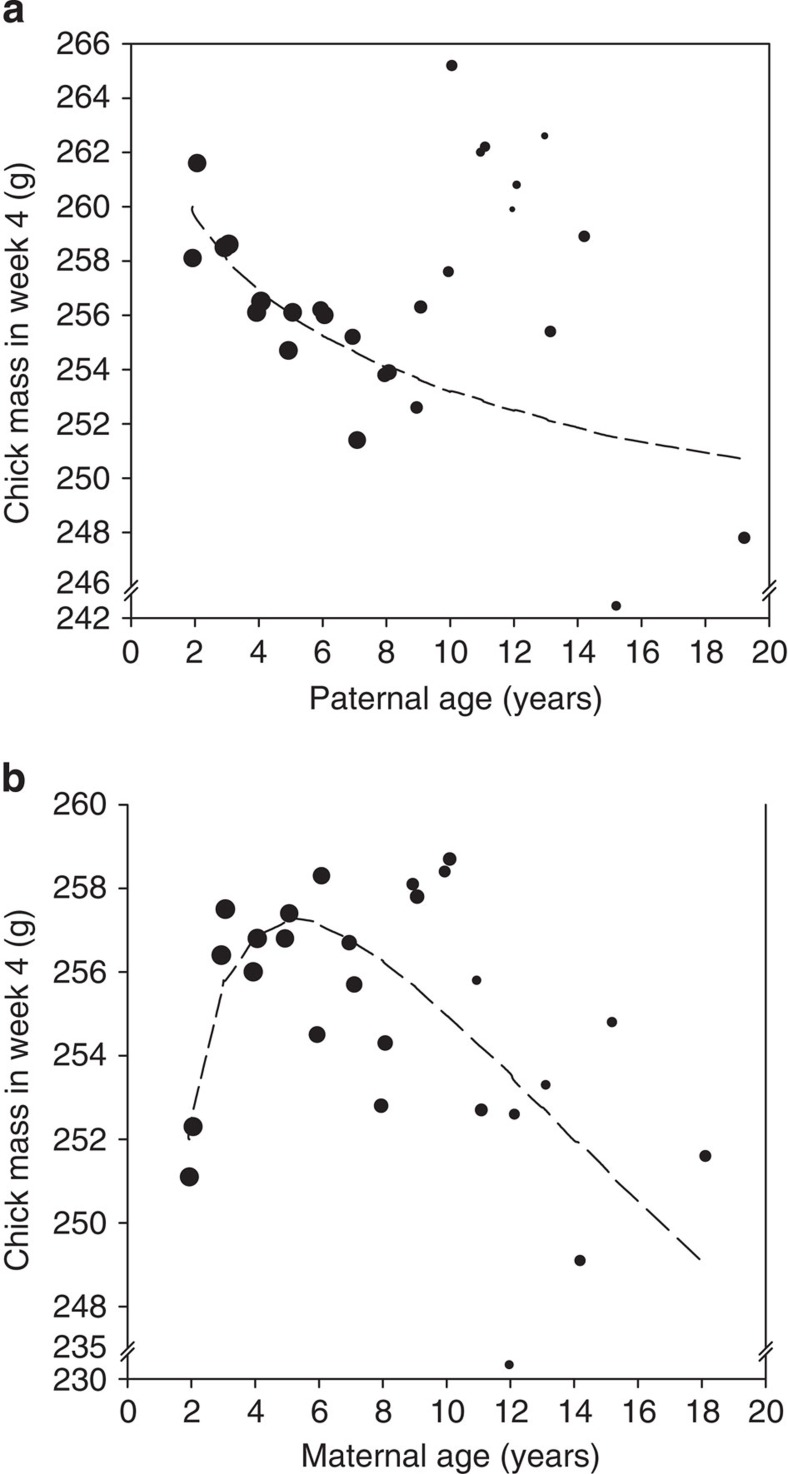
Sex differences in the influence of parental senescence on offspring mass. The plots show how (**a**) paternal age and (**b**) maternal age have differing consequences to the mass of their offspring as measured in their fourth week of development. The data are grouped at intervals of 6 months up to 16 years of age after which all remaining data are grouped due to reduced availability of sample. The sample available in each grouping is reflected by size of the data points (logged).

Compensatory mechanisms may influence the growth of poor quality chicks post hatching, dampening early variation in offspring mass (catch-up growth^18^) and potentially confounding analyses. Thus, to investigate directly whether the gametes of aging parents actually yield offspring of lower body mass, we implemented a new analysis examining only a single measurement of each chick's mass that was collected in the fourth week of development. ‘Chick mass at hatching' was specifically excluded from this analysis, and thus in contrast to the analysis presented immediately above this analysis examines the overall influence that maternal and paternal age could contribute to their offspring's growth at the end of their first month of life. We found that the effect of paternal age on chick's early growth was echoed in their mass after a month, with the youngest fathers producing the heaviest offspring (Fig. 4a). Since the youngest males produce spermatids that are on average of poor functional quality^12^, this relationship is more consistent with expectations if mutation-based aging of a male's germ line is hindering their offspring's performance, rather than it being a consequence of the declining functional performance of their sperm. As might be expected, we also found that the overall influence of maternal age on their offspring's early development mirrored those found in both their likelihood of hatching successfully and their body mass at hatching, such that mothers that are at their ‘peak ages' of 3 to 6 years produced the heaviest offspring after a month of life (see Table 4; Fig. 4b). As we observed little overall influence of maternal age on their offspring's growth after chicks had hatched, we postulated that differences associated with maternal age might be generated predominately through the ability of mothers to provision their eggs. To test this possibility, we included ‘egg mass' as a term in this analysis, using it as a proxy for egg provisioning. We found a strong positive relationship between egg mass and offspring mass after a month of life (LMM: estimate=1.351; s.e.=0.0673; Wald statistic=402.58; degree of freedom=1; *n*_eggs_=18109; *P*<0.001), while maternal age became a non-significant term in this analysis (LMM: *n*_eggs_=18109; *P*>0.24), suggesting that, as hypothesized, the influence of maternal age was mediated through an increased ability of ‘peak age' females to provision their eggs.

## Discussion

We found that aging houbara bustards experience decline in their ability to sire high quality offspring. For both sexes, aging beyond a ‘prime' of around 3 to 6 years of age was associated with an increase in the number or frequency of eggs that failed to hatch, while reduced growth rates experienced by their progeny, either pre or post hatching depending on the sex of the parent, meant chicks were significantly lighter at 1 month of age. For females, any shortfall in offspring growth occurred while they developed within eggs, and our analysis is consistent with retarded growth being explained by a reduced ability of older females to provision their eggs with the nutrients required for greater zygote development^14^. This mechanism may also explain the declining hatching success of eggs from older (and younger) females^19^. For aging males, however, the correlated decline in the growth rate of progeny occurred only post hatching. Since males contribute just DNA to their offspring, and females were unaware of paternal identity, it would appear that chick growth was inhibited by an age-related decline in the quality of male germ line DNA. Two further lines of evidence are consistent with this interpretation. First, the build-up of germ line mutations that could result in this age-related degradation of DNA within spermatozoa would be expected to occur from the point at which the male germ line first begins replicating^10^,^11^, a theoretical expectation that is met by our finding that growth rates are highest in chicks sired by the youngest males. The superiority of immature males in this respect is in marked contrast to their egg hatching success. Here young males experience lower success relative to ‘prime' aged animals in a manner that is consistent with both the observed maturational increase in the number and quality of spermatozoa produced by their spermatogenic machinery (prior to later senescent declines^12^), and also approximates changes in the size and hatching success of female gametes across life that is observed here. Second, the absence of an equivalent effect of maternal aging on the post-hatching growth rate of progeny is also consistent with germ line DNA senescence. Germ line replication rates, and hence the build-up of genetic mutations within gametic DNA, are far greater in males than females due to the requirement for males to produce many millions of gametes to successfully fertilize a single egg, particularly in species in which sperm competition is prevalent^11^,^20^. Thus, overall, it would appear that the functional performance of spermatozoa and the integrity of the DNA they carry suffer senescent declines as males age, leading to reductions in the viability and quality of their offspring.

Our results suggest that egg hatching failure resulting from age-related declines in the performance of male ejaculates could represent a significant reproductive cost to females in the wild. Our data suggest that males 10 years past their prime constitute an additional 7% risk of reproductive failure per egg for females inseminated by them. As the conservation programme uses artificial insemination of females for fertilization, some degree of caution should be employed when applying these estimates of reproductive decline in aging males to the natural condition. However, it should also be noted that the magnitude of these reductions are likely to have been attenuated by the quality control measures that are implemented by the conservation programme. Poor quality ejaculates (<50% motility) will not be used to inseminate females and older males routinely produce ejaculates of poor quality (∼65% of ejaculates for 14 year old males^12^), thus, the implementation of a quality selection criterion is likely to have artificially shifted their hatching success markedly upwards relative to peak aged males, whose ejaculates are of consistently higher quality. In other words, older males may obtain greater benefit from the ‘weeding out' of poor ejaculates as they produce them more often, and as a result, our estimate of reproductive failure owing to increasing male age is likely to be conservative. This mechanism assumes that the measures of quality used for ejaculate selection are related to viability of eggs, which may not be the case, particularly where hatching failure is due to embryo death and not male infertility.

Adult houbara bustards have an 80–90% annual survival rate in the wild, and can live until at least 23 years of age in captivity, suggesting that older males with impaired germ line DNA are likely to be present in the breeding population. Assuming similar age-related reductions in male fertility occur in the wild and cannot be reliably detected by females, this could provide a selective pressure favouring female solicitation of repeated copulations from their mates, or seeking extra pair copulations, as direct fertility benefits may result from receiving additional functional sperm^21^,^22^. Accordingly, female houbara bustards typically mate promiscuously, with 60% of their clutches having multiple sires^23^.

In addition, our results show for the first time that senescence of gametic DNA from aging males leads to a quantifiable reduction in key indicators of their offspring's ‘quality'. Chicks of males that were 14 years of age produced chicks that were 3% lighter a month after hatching when compared with offspring they produced at the onset of spermatogensis, which is of similar magnitude to the declines experienced by the chicks of older mothers from their peak age with respect to offspring growth (a 2.5% decline in offspring body mass for females aging from 4 to 14 years of age). Given the influence that maternal egg provisioning can have on offspring performance^24^, and the decline in provisioning from aging females found here and in other studies^14^, quantifiable reductions in offspring performance with maternal senescence are to be expected. However, it is surprising to find that there is a similar, or greater, effect of paternal aging on offspring performance, which appears to be a consequence of senescent effects on male's gametic DNA.

As with egg viability, conditions within the programme seem likely to have led to our study underestimating the true impact of male aging on offspring quality. Chicks are hand reared by the programme, provided with a ready supply of high quality feed and sheltered from harsh environmental conditions. Chicks would thus be largely isolated from any negative influence of sibling competition, for example, ref. 25, or the need to cope with harsh environmental conditions, for example, ref. 26, which would otherwise be expected to exacerbate the already reduced growth rate of poor quality chicks sired by older males. Thus, while we find evidence of a significant cost to offspring quality associated with paternal senescence in male houbara bustards, our analyses seem certain to underestimate the true cost of mating with an old male in the wild.

Patterns found in humans are congruent with our findings in houbara bustards and their interpretation. A recent study examining genome-wide mutations in humans showed that the occurrence of mutations in gametic DNA was strongly associated with paternal rather than maternal age, with an estimated two new mutations occurring with every additional year of paternal aging^27^. In assessments of the consequences of sperm DNA ‘damage' using *in vitro* fertilization studies, negative effects have been found on pregnancy rates, embryo development and the number of live births^28^,^29^, pointing towards the potential for a similar cost of sperm DNA ‘damage' caused by age-related mutation. More generally, increasing paternal age in humans has also been associated with adverse reproductive outcomes, as well as a number of genetic diseases and mental disorders in offspring, prompting heightened concern over the trend for delaying parenthood until later in life^9^. This study has deepened our understanding of these concerns by providing evidence for a mechanistic link between paternal aging and offspring health.

Until now, a number of factors have precluded our ability to identify and subsequently quantify reproductive consequences of paternal aging in a wild long-lived species^5^. Aside from the difficulty in generating sufficient longitudinal data on male reproduction as they age, progress has been hindered by inherent difficulties in separating the effects of gametic performance from the expectation that females will vary investment in the sperm or offspring of males based on their perceived quality^30^, which would be expected to decline with male age^6^,^8^. Similarly, in species with male paternal care, the quality of care is expected to covary with male age, thus confounding potential relationships with age-related changes in gamete quality^31^,^32^. Here neither parent provides care to offspring beyond their gametes, nor can they choose or have knowledge of the other parent's identity or age, and therefore age-related changes in the viability and quality of male offspring can be unambiguously explained as being a result of senescent effects on their sperm.

Our findings that there are significant direct and indirect reproductive costs to females when mating with older males have implications for the age-based indicator theory of sexual selection. This idea suggests that females will gain indirect genetic benefits from older males that have demonstrated their ability to survive in current environmental conditions, and they should thus develop mating preferences for them^33^,^34^. However, our results suggest that the benefits to females of choosing male genotypes that have high survival probability would have to be substantial to offset both the direct and indirect costs found here of using their senescing sperm to fertilize ova. In some species, the indicator traits of males on which females may base their choice also appear to decline in old age, for example, refs 6, 8, perhaps maintaining a more reliable link between their inherent genetic quality and the functional performance of their sperm^35^. This does not appear to be the case for male houbara bustards, however, as there is little evidence of a senescent decline in their extravagant sexual display as they age, even while their ejaculate quality and sperm viability undergoes sharp declines^12^. Indeed, our results suggest that any indirect ‘good genes' benefits to be gained from males that express age-related traits may be eroded through the senescence of their germ line DNA even as these traits are developing^11^.

Surprisingly, in terms of offspring growth, it is the youngest houbara male that appears to offer the greatest indirect reproductive benefit to females, though this benefit would be countered by the poor hatching success of their eggs. Assuming this poorer hatching success is due to their sperm failing to fertilize eggs, as would be suggested by the poor overall quality of their ejaculates^12^, then this limitation of young males could potentially be overcome if females mated with additional males as a means to assure fertilization^21^,^22^. This may explain why females of various species will sometimes engage in promiscuous matings with younger subordinate males^36^,^37^, when the risk of fertilization failure has been ameliorated through copulations with mature individuals. Indeed, the relatively poor hatching success of both younger and older males would seem likely to favour the evolution of promiscuous mating behaviour in females if by doing so females can insure against a cost of large age-related changes in male fertility^10^. Since male-biased mutation rates (and so germ line senescence) are driven in part by the requirement for high sperm production rates, driven themselves by the need to produce large number of sperms under conditions of sperm competition^11^,^21^, it may be that female promiscuity also drives female mating preferences towards younger males.

## Methods

### Housing and rearing conditions

All birds considered here were part of a large-scale captive breeding programme located in Eastern Morocco^38^. Birds originated from the programme or were collected as eggs from the wild and were thus of known age^39^. Parents were housed in three separate locations (Almis, Missour and Enjil), but were reared and held under similar conditions with *ad libitum* access to food and water. All eggs were removed from females soon after being laid and were incubated in Missour at ∼37.5 °C, 12 to 55% humidity prior to hatching. Hatched chicks were then intensively hand reared indoors for the first 10 days, before being transferred to outdoor cages (4 m^2^ in size) in groups of 6 unrelated individuals, where food and water were available *ad libitum* for the remaining period under investigation here. Mortality rate within this first month was <5%.

Procedures complied with ethical regulations and were approved by the ‘Ministère de l'Agriculture, Développement Rural et des Pêches Maritimes, Direction Provinciale de l'Agriculture de Boulemane, Service Vétérinaire' (Nu DPA/48/285/SV) under permit N° 01-16/VV; OAC/2007/E; Ac/Ou/Rn.

### Artificial insemination

Ejaculates were collected from donor males according to the needs of the captive breeding programme, but allowing at least 1 day of recovery between collections in 97% of cases. Collections were performed using a dummy female, which allowed males to exhibit the full range of copulatory behaviour prior to ejaculation. The samples collected were then rapidly screened for quality in an adjoining laboratory, including counts of sperm number and an assessment of motility. A detailed description of the collection methodology and frequencies is available elsewhere^12^,^40^. Ejaculates used by the programme generally contained >10 × 10^6^ sperm (>97% cases) and were at least 50% motile. These ejaculates underwent appropriate dilution with Lake 7.1 diluent^41^ and were used to inseminate females the same morning according to standardized protocols (see ref. 40). Briefly, the female's cloaca was opened using retractors and assessed for reproductive state (based on characteristics of the cloaca, vagina and ischium^40^). For selected females, a straw was affixed to a plastic syringe containing the semen and inserted into the proximal vagina (at ∼4cm depth) before the sperm was slowly introduced. Inseminations comprised an average of 15 × 10^6^±7.452 sperm (average±s.d.) within 80 μl±38.32 (average±s.d.) of semen/diluent. Females were subsequently checked each morning and the eggs (if any) were collected for incubation.

### Statistical analysis

The analyses were performed using GLMM or LMM in the Genstat statistical package (12th edition) and using all relevant data collected by the programme. Mixed models are powerful tools that allow the analysis of longitudinal data on individuals to examine the changes that occur with age by fitting the identity of individuals as a random effect^15^,^42^. Our central analyses here use longitudinal measurements collected over 10 years (from 2000 to 2009), of both the hatching success of eggs as prospective parents become older (mean=22.9 eggs per mother and 50.8 eggs per father), and repeated measurements of the growth of successfully hatched offspring during their first month of life (mean=13.3 measurements per chick). Potential parents ranged from 1 to 23 years of age. Specific sample sizes vary according to the availability of data, and are provided in the legends of tables reporting analyses or associated with statistics in the main text.

In analyses of hatching success and chick mass, the identity of parents and the year of breeding were controlled as categorical random effects^43^. Chick identity was also included as a random effect in the analysis examining chick growth. ‘Day of year' was fitted as a cubic-fixed term in models to test for cyclical seasonal changes in hatching success and growth. Females laid eggs in a median of 2 per clutch (range 1 to 19, eggs laid within 3 days of each other were defined as belonging to the same clutch), and produced a median of 4 clutches per year (range 1 to 15). Declining quality of eggs within and between clutches, and thus potentially both hatching success and chick mass, was controlled for by testing ‘clutch number' and ‘within clutch order' as quadratic fixed terms in models.

The age of individuals was calculated for the day on which the ejaculates, eggs or measurements were collected. Adult age was fitted into models as a quadratic function to allow for the curvilinear relationship between age and reproductive parameters that was apparent in the raw data and has previously been described for male ejaculate characteristics in this species^12^. Offspring growth trajectories within their first month of development were well-described using a cubic polynomial. For the analysis of chick growth rates, parental ages were fitted in interaction with offspring age (all terms). This allowed the model the freedom to detect changes in the influence (and direction) of parental age on offspring growth as chicks became older.

During analyses, systematic departure from the model fit was observed for individual's ages and the clutch parameters; this was corrected through log_e_ transformation of these terms. The final models that are reported in tables had non-significant (*P*>0.05) terms removed^44^. The fitted lines presented in plots are derived from model predictions, and the data are controlled for other terms included in the model. Re-analyzing our models using mean centred data produced qualitatively similar results and predictions in all cases.

## Author contributions

B.T.P., M.S.J., Y.H., F.L. and G.S. conceived the study; Y.H. and F.L. supervised and participated in data collection; B.T.P. designed and performed the analysis with G.S.; B.T.P. wrote the first draft of the manuscript and all authors contributed to revisions.

## Additional information

**How to cite this article**: Preston, B. T. *et al.* The sperm of aging male bustards retards their offspring's development. *Nat. Commun.* 6:6146 doi: 10.1038/ncomms7146 (2015).

## Figures and Tables

**Table 1:** GLMM of the influence of parental age on egg hatching success.

**Term**	**d.f.**	**Effect**	**s.e.**	**Wald stat (*****χ***^2^**)**	***P*** **value**
Day	1	0.01144	0.005035	5.16	0.023
Day^2^	1	−0.00009522	0.000036212	6.91	0.009
Day^3^	1	0.0000001941	8.1796E−08	5.63	0.018
Clutch number	1	0.2659	0.04786	30.88	<0.001
Clutch number^2^	1	−0.09423	0.027588	11.67	<0.001
Within clutch order	1	0.1597	0.06327	6.37	0.012
Within clutch order^2^	1	−0.2615	0.06610	15.65	<0.001
Maternal age	1	4.083	0.6218	43.13	<0.001
Maternal age^2^	1	−0.2845	0.04296	43.87	<0.001
Paternal age	1	1.587	0.5048	9.88	0.002
Paternal age^2^	1	−0.1089	0.03483	9.78	0.002

d.f., degree of freedom; GLMM, Generalized Linear Mixed Model; s.e., standard error.

The aim of the analysis was to examine the potential influence of parental aging on the hatching success of their eggs. A binary variable was used which indicated whether eggs that had been incubated subsequently hatched. A logistic GLMM was implemented using a logit link function; constant=−20.06. In total, the analysis includes assessments of 58,977 eggs laid by a total of 2,580 females and fertilized by a total of 1,161 different males. Parental identity and year of breeding were controlled as random effects. Variables derived from parental age and clutch sequences data were log_e_ transformed to improve model fit.

Numbers in superscript indicate that the terms have been fitted as a second/third order polynomial.

**Table 2:** LMM of the influence of parental age on chick mass at hatching.

**Term**	**d.f.**	**Effect**	**s.e.**	**Wald stat (*****χ***^**2**^**)**	***P*** **value**
Day	1	−0.008883	0.0010602	70.19	<0.001
Clutch number	1	−0.9716	0.04027	582.10	<0.001
Within clutch order	1	−4.175	0.0929	2021.61	<0.001
within clutch order^2^	1	1.518	0.0995	232.89	<0.001
Maternal age	1	33.12	1.172	798.67	<0.001
Maternal age^2^	1	−2.245	0.0841	712.17	<0.001
Paternal age	1	1.334	0.7418	3.23	0.072
Paternal age^2^	1	−0.08959	0.051134	3.07	0.080

d.f., degree of freedom; LMM, Linear Mixed Model; s.e., standard error.

The aim of the analysis was to examine the potential influence of parental aging on the hatching mass of their offspring. A LMM was implemented with chick mass at hatching as the response variable; constant=−84.06. In total, the analysis includes assessments of 41,844 chicks produced by 2,488 females and fertilized by 1,140 different males. Parental identity and year of breeding were controlled as random effects. Variables derived from parental age and clutch sequences data were log_e_ transformed to improve model fit.

Numbers in superscript indicate that the terms have been fitted as a second order polynomial.

**Table 3:** LMM of the influence of parental age on offspring growth.

**Term**	**d.f.**	**Effect**	**s.e.**	**Wald stat (*****χ***^**2**^**)**	***P*** **value**
Chick mass	1	1.286	0.0241	2844.66	<0.001
Day	1	−0.7757	0.005035	352.00	<0.001
Day^2^	1	0.003950	0.000036212	192.58	<0.001
Day^3^	1	−0.000004450	0.0000006178	51.86	<0.001
Chick age	1	1060	82.3	—	—
Chick age^2^	1	−699.8	50.5	—	—
Chick age^3^	1	163.1	9.19	—	—
Maternal age	1	110.8	10.34	—	—
Maternal age^2^	1	−7.386	0.7003	—	—
Paternal age	1	113.4	9.27	—	—
Paternal age^2^	1	−7.503	0.6323	—	—
Maternal age × Chick age	1	−122.3	17.33	49.83	<0.001
Maternal age^2^ × Chick age	1	−5.332	0.7114	49.50	<0.001
Maternal age × Chick age^2^	1	78.89	10.514	56.31	<0.001
Maternal age^2^ × Chick age^2^	1	−5.332	0.7114	56.18	<0.001
Maternal age × Chick age^3^	1	−17.42	1.899	84.14	<0.001
Maternal age^2^ × Chick age^3^	1	1.177	0.1286	83.69	<0.001
Paternal age × Chick age	1	−156.3	15.06	107.72	<0.001
Paternal age^2^ × Chick age	1	10.46	1.021	104.96	<0.001
Paternal age × Chick age^2^	1	101.9	9.38	117.89	<0.001
Paternal age^2^ × Chick age^2^	1	−6.807	0.6370	114.22	<0.001
Paternal age × Chick age^3^	1	−22.72	1.723	173.92	<0.001
Paternal age^2^ × Chick age^3^	1	1.503	0.1170	164.99	<0.001

d.f., degree of freedom; LMM, Linear Mixed Model; s.e., standard error.

The aim of the analysis was to examine the potential influence of parental aging on the early growth of their offspring. Repeated measurements of chick growth within the first month were used as the response variable and a LMM was implemented; constant=−813.6. In total, the analysis includes 493,700 measurements of 31,404 chick's masses belonging to 2,330 females and fertilized by a total of 1,074 different males. Note that chick identity was controlled in this model as a random effect, in addition to parental identity and year of breeding, to account for the use of repeated measures of chick mass within the analysis. Parental and chick age were log_e_ transformed to improve model fit.

Numbers in superscript indicate that the terms have been fitted as a second/third order polynomial.

**Table 4:** LMM of the influence of parental age on offspring mass.

**Term**	**d.f.**	**Effect**	**s.e.**	**Wald stat (*****χ***^**2**^**)**	***P*** **value**
Day	1	−3.043	0.148	425.65	<0.001
Day^2^	1	0.0165	0.001	272.02	<0.001
Day^3^	1	−0.0000231	0.00000207	124.40	<0.001
Chick age	1	−1772	363.4	23.77	<0.001
Chick age^2^	1	317	56.67	31.28	<0.001
Maternal age	1	80.88	19.679	16.89	<0.001
Maternal age^2^	1	−5.357	1.348	15.79	<0.001
Paternal age	1	−4.0140	0.997	16.23	<0.001

d.f., degree of freedom; LMM, Linear Mixed Model; s.e., standard error.

The aim of the analysis was to examine the potential influence of parental aging on the mass of their offspring in their fourth week of development. An LMM analysis was implemented using one measurement of chick mass from the fourth week (chosen at random) as the response variable; constant=2,568. In total, the analysis includes 18,109 chicks with 2,029 mothers and 939 different sires. Parental identity and year of breeding were controlled as random effects. Parental and chick age were log_e_ transformed to improve model fit.

Numbers in superscript indicate that the terms have been fitted as a second/third order polynomial.
